# Rhamnolipids: solution against *Aedes aegypti*?

**DOI:** 10.3389/fmicb.2015.00088

**Published:** 2015-02-16

**Authors:** Vinicius L. Silva, Roberta B. Lovaglio, Claudio J. Von Zuben, Jonas Contiero

**Affiliations:** ^1^Biochemistry and Microbiology, Universidade Estadual Paulista Júlio de Mesquita FilhoRio Claro, Brazil; ^2^Zoology and Entomology, Universidade Estadual Paulista Júlio de Mesquita FilhoRio Claro, Brazil

**Keywords:** biosurfactant, entomology, *Pseudomonas aeruginosa*, tropical diseases, repellent

## Abstract

*Aedes aegypti* mosquitoes are the primary transmitters of dengue fever, urban yellow fever, and chikungunya viruses. This mosquito has developed resistance to the insecticides currently used to control their populations. These chemical insecticides are harmful to the environment and can have negative effects on human health. Rhamnolipids are environmentally compatible biological surfactants, but their insecticidal activity has not been extensively studied. The present study evaluated the potential larvicidal, insecticidal, and repellent activities of rhamnolipids against *A. aegypti*. At concentrations of 800, 900, and 1000 mg/L, rhamnolipids eliminated all mosquito larvae in 18 h and killed 100% of adults at 1000 mg/L. According to the results it may be conclude that rhamnolipids should be applied to control larvae and mosquitos besides present the repellency activity against *A. aegypti*.

## INTRODUCTION

Rhamnolipids are biological surfactants that exhibit low toxicity and high biodegradability with few adverse effects on the environment. Environmental compatibility associated with the use of renewable carbon sources and production by microorganisms makes these compounds excellent substitutes for chemical surfactants given that both have similar physico-chemical properties (Desai and Banat, 1997; Banat et al., 2000; Rahman et al., 2010; Sekhon Randhawa and Rahman, 2014). Due to their capacity to reduce surface and interfacial tension, application of these microbial compounds in a broad range of industrial sectors has been proven and new applications have been evaluated (Rahman and Gakpe, 2008; Vatsa et al., 2010).

Kim et al. (2011) demonstrated the potential of rhamnolipids produced by *Pseudomonas aeruginosa* as an insecticide against aphids. These microbial metabolites are also efficient against *Rhyzopertha dominica*, a beetle species that attacks stored grains (Kamal et al., 2012). Biosurfactants produced by microbes can be used to control pests (Awada et al., 2005).

Substitution of insecticides by compounds with low toxicities such as rhamnolipids may contribute to reduced environmental and social impacts compared to chemicals. Furthermore, these compounds are an alternative to resistance developed by a significant number of arbovirus vectors (arthropod-borne viruses).

*Aedes aegypti* is anthropophilic and endophilic mosquito species. The larvae use tracheal breathing, and their respiratory siphon is located at the posterior of their body. For gas exchange, they must remain in an angle close to 90^∘^ with the surface of the water (±20^∘^) depending on larval stage (Christophers, 1960).

This culicid species is of significant medical importance because it is the primary vector of urban yellow fever, dengue fever and chikungunya (Eldridge, 2005; Vontas et al., 2012).

According to Tsai et al. (2013), 40% of the worldwide population is at risk of contracting dengue fever, rendering this disease one of the main arboviruses transmitted from mosquitoes to humans. An estimated 50–100 million cases of dengue fever occur worldwide per year (Kroeger and Nathan, 2006; Kourí et al., 2007; Garelli et al., 2013). These viruses are responsible for 30,000 deaths per year (World Health Organization, 2009).

Due to globalization, rampant population growth and climate change, it is likely that areas previously not know for dengue fever may become suitable sites for disease occurrence. After more than a half-century without a dengue outbreak in the United States, there have been recent outbreaks in Texas (2004–2005) and Florida (2009–2011) (Eisen and Moore, 2013). Rogers et al. (2014) reported a risk of dengue in some areas of Europe.

The use of chemical insecticides to control insect vectors is contrary to the recent global focus on developing products with low environmental impacts. Although population control is currently achieved using temephos for larvae and pyrethroids for adult mosquitoes (Chavasse and Yap, 1997), resistance to these compounds has been observed.

Potential negative effects of using chemical insecticides have led to research and development of products that are less harmful to the environment (Mittal, 2003). Biological pesticides produced by bacteria are effective against mosquitoes at low doses and do not affect other biological control agents (Walton and Mulla, 1992). This study evaluated the larvicidal, insecticidal, and repellent activities of rhamnolipids to verify their potential use as substitutes for insecticides that are currently used for *A. aegypti* control.

## MATERIALS AND METHODS

### RHAMNOLIPIDS PRODUCTION

The *P. aeruginosa* strain LBI 2A1 was used to produce rhamnolipids. Culture media and growth conditions were previously described by Müller et al. (2010). Fermentation using sunflower oil as a carbon source was performed in a bioreactor. Rhamnolipids isolated from sunflower oil were used in the experiments.

### RHAMNOLIPIDS EXTRACTION

Fermented broth was centrifuged at 4000 rpm for 30 min, and equal volumes of cell-free supernatant and n-hexane were thoroughly mixed in a volumetric flask. The mixture was then allowed to settle until the organic and aqueous phases separated. The organic phase was removed and 85% H_3_PO_4_ 1:100 (v/v) was added to the aqueous phase to precipitate rhamnolipids. Biosurfactants were extracted with ethyl acetate 1:1.25 (v/v). The mixture was shaken for 10 min, allowed to settle, and the upper phase was removed. This extraction process was repeated using the lower phase extract. Extracted rhamnolipids were concentrated using a rotary evaporator, and the viscous yellowish product was dissolved in methanol and concentrated again by evaporation of residual solvent at 45^∘^C.

### STATISTICAL ANALYSIS

Data were compared by one-way analysis of variance followed by quadratic regression test when significant differences were found at *P* = 0.05 (Sokal and Rohlf, 1995). The software used was Sisvar 5.3.

### MOSQUITO AND LARVAL MAINTENANCE

The Center of Zoonosis Control (Santa Bárbara D’Oeste, São Paulo, Brazil) provided the parental *A. aegypti* specimens. The mosquitoes were maintained in plastic cages in a room with controlled temperature at 27 ± 2^∘^C and a photoperiod of 12 h. Adults were fed 10% glucose w/v.

For egg and larval acquisitions, blood feeding was performed using a laboratory mouse sedated with ketamine (approved by the Ethics Committee on Animal Use, Protocol n^∘^: 3698, Decision from CEUA N^∘^ 022/2011). After the blood feeding, 600 ml flasks with 300 ml of mineral water were placed in the cages. A 12 × 4 cm wood palette was used for oviposition. Flasks were filled with water until eggs were submerged. Larval feeding was performed with fish food (Löwenberg Neto and Navarro-Silva, 2004).

### LARVICIDAL EVALUATION

A total of 40 ml of either rhamnolipids or control solution with 10 *A. aegypti* larvae in the third instar of development were added to 60 ml flasks. Small holes in the flask caps allowed for gas exchange. Control (mineral water) and solutions containing 50, 100, 200, 300, 400, 600, 800, 900, and 1000 mg/L rhamnolipids were evaluated. Experiments were performed in duplicate. Larvicidal activity was quantified based on time of permanence at the surface and the bottom and the numbers of attempts to stay on the surface, these factors were quantified in real time observations at intervals of 3 or 6 h. The larvae were considered dead when they did not show any sign of activity and movement for more than 3 h.

### REPELLENCY EVALUATION

The following rhamnolipids concentrations, which demonstrated optimal performance in larvicidal evaluation, were used to evaluate repellency: 400, 800, and 1000 mg/L. Mice were sprayed with 2,400μL of the solutions and labeled according to the type of treatment. Each cage contained a mouse for each treatment and a total of 25 mosquitoes, with an observation time of 40 min. The number of mosquitoes that landed on each mouse was counted as host attraction. Tests were performed four times, and the mosquitoes’ activity was quantified in real time by the researchers.

### INSECTICIDAL EVALUATION

The concentrations evaluated for insecticidal activity were the same as in the repellency test. Experiments were conducted in triplicate, with each cage containing 20 mosquitoes and was sprayed whit 3,200 μL of rhamnolipids solution. Dead mosquitoes from each treatment were chosen randomly, for analysis in optical microscopy.

## RESULTS

### LARVICIDAL ACTIVITY

**FIGURE 1:**
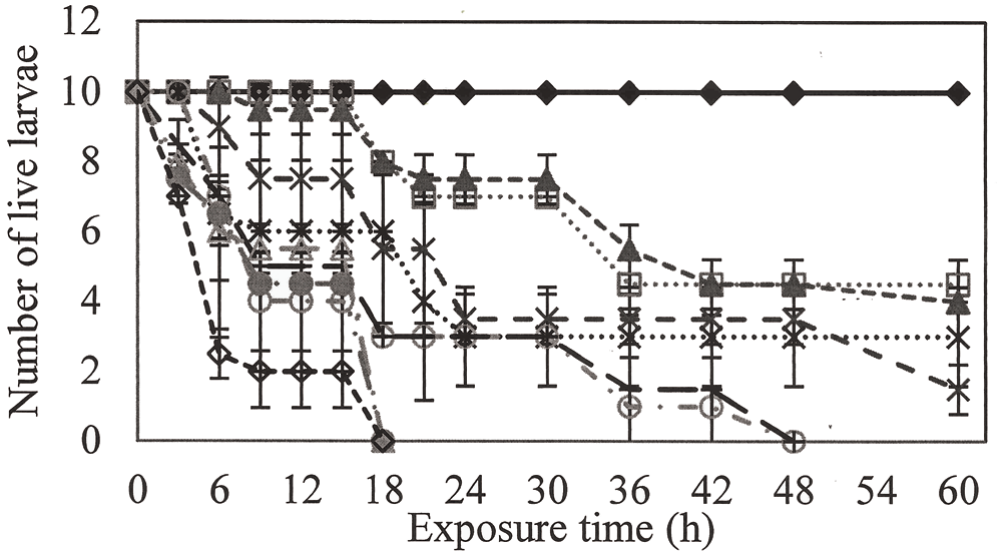
**Number of live larvae over time when exposed to different concentrations of rhamnolipids.** Ten larvae of Aedes aegypti were exposed to: Control (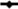), 50(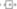), 100(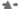), 200(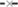), 300 (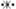), 400(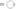), 600(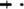), 800(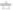), 900(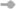) e 1000(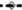) mg/L of rhamnolipids.

Larvicidal activity was observed at all rhamnolipids concentrations evaluated, with a minimum mortality rate of 50%. Rhamnolipids concentrations of 800, 900, and 1000 mg/L killed all larvae at 18 h (*P* = 0.00; *R*^2^ = 0.92), while 400 and 600 mg/L solutions required exposure for 48 h to reach 100% mortality (*P* = 0.00; *R*^2^ = 0.86; **Figure 1**).

**FIGURE 2:**
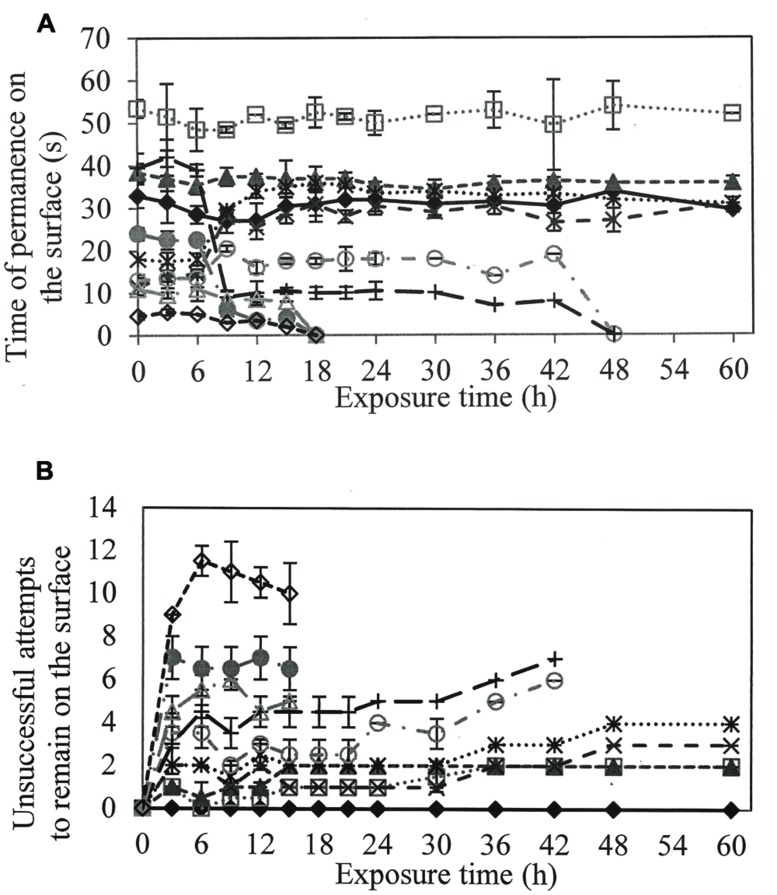
**(A)** Time of permanence on the surface of the larvae to perform the gas exchange and **(B)** Number of unsuccessful attempts of the larvae in remain on the surface, when in contact with different solutions of rhamnolipids : Control (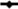), 50(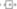), 100(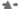), 200(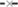), 300 (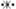), 400(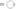), 600(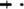), 800(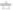), 900(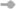) e 1000(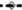) mg/L of rhamnolipids.

To evaluate if larvicidal activity was related to a reduction in surface tension and respiratory activity of the larvae, the residence time on the surface and the number of unsuccessful attempts to remain at the interface of the water surface were quantified (**Figure 2A**).

In solutions containing 50 and 100 mg/L rhamnolipids, larvae tended to stay longer on the surface compared to the control treatment. However, the larvae required more than one attempt to remain on the surface in the correct position to perform gas exchange (**Figure 2B**).

At concentrations greater than 400 mg/L, length of time on the surface was shorter than observed in controls. The number of attempts to settle on the surface increased proportionally to rhamnolipids concentration.

**FIGURE 3:**
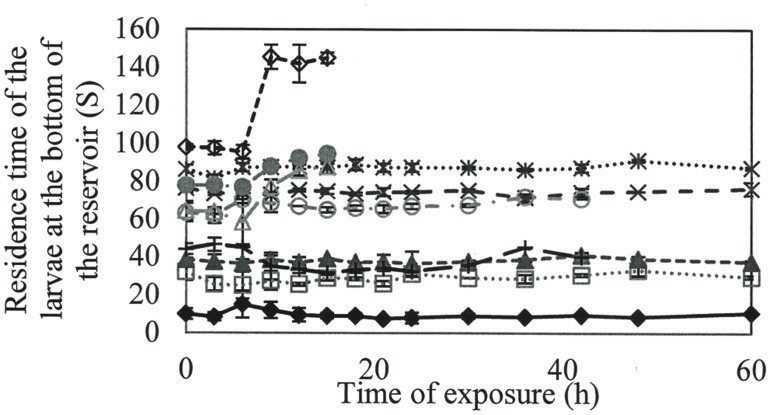
**Time that the A. aegypti larvae remained in the bottom of the reservoir along the time when exposed to different concentrations of rhamnolipids: Control (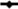), 50(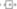), 100(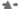), 200(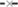), 300 (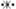), 400(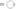), 600(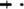), 800(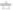), 900(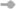) e 1000(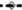) mg/L of rhamnolipids**.

To verify the loss of larval activity over time, the time of larval permanence at the bottom of the flask was quantified (**Figure 3**).

Larvae in the control group had the lowest length of time at the bottom of the flask, with an average of 30.6 s. At concentrations of 800–1000 mg/L, the hydrostatic balance was broken between the water and larvae, as evidenced by an increase in the length of time at the bottom, culminating in death after 18 h.

### REPELLENCY

**FIGURE 4:**
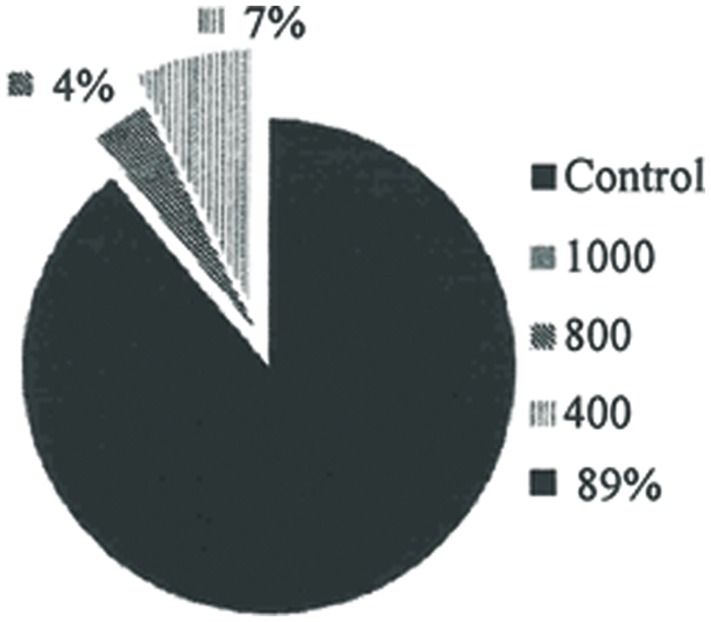
**Percentage of mosquitoes that landed on the mice and practiced hematophagy, subjected to different solutions of rhamnolipids: Control, 400, 800 e 1000 mg/L of rhamnolipids (P = 0.00; R2 = 0.97; all concentrations evaluated were significantly different at P = 0.05)**.

The repellent property of rhamnolipids produced by *P. aeruginosa* was demonstrated given that mosquitoes had a higher incidence of landing on mice in the control group. The host attraction attempts were reduced with increased concentrations of the biosurfactant (**Figure 4**).

Another factor evaluated was the time spent feeding by mosquitoes at different rhamnolipids concentrations (**Table 1**).

**Table 1:** Average time of hematophagy on mice treated with different rhamnolipids concentrations: control, 400, 800, and 1000 mg/L (*P* = 0.00; *R*^2^ = 0.99).

Rhamnolipids concentration (mg.L-^1^)	Average length of stay on the mice (*s*)	*SD*
Control	23.08 (a)*	1.65
400	5.15 (b)	3.28
800	1.18 (c)	1.36
1000	0.00 (d)	0.00

According to the results, the microbial metabolite produced by *P. aeruginosa* appears to reduce the residence time spent by mosquitoes in addition to reducing the total number of mosquitoes.

### Insecticidal effects

The insecticidal activity of rhamnolipids against mosquitoes showed a 100% elimination of adults at the highest concentration evaluated compared to only 8% in the control group (**Table 2**).

**Table 2:** Number of live *Aedes aegypti* adults after application of different rhamnolipids concentrations: control, 400, 800, and 100 mg/L.

Rhamnolipids concentration (mg.L-^1^)	Initial number of mosquitoes	Average number of live mosquitoes	Deviation	% of elimination
Control	20	18.33	1.53	8
400	20	2.67	0.58	87
800	20	1.33	0.58	93
1000	20	0.00	0.00	100

**FIGURE 5:**
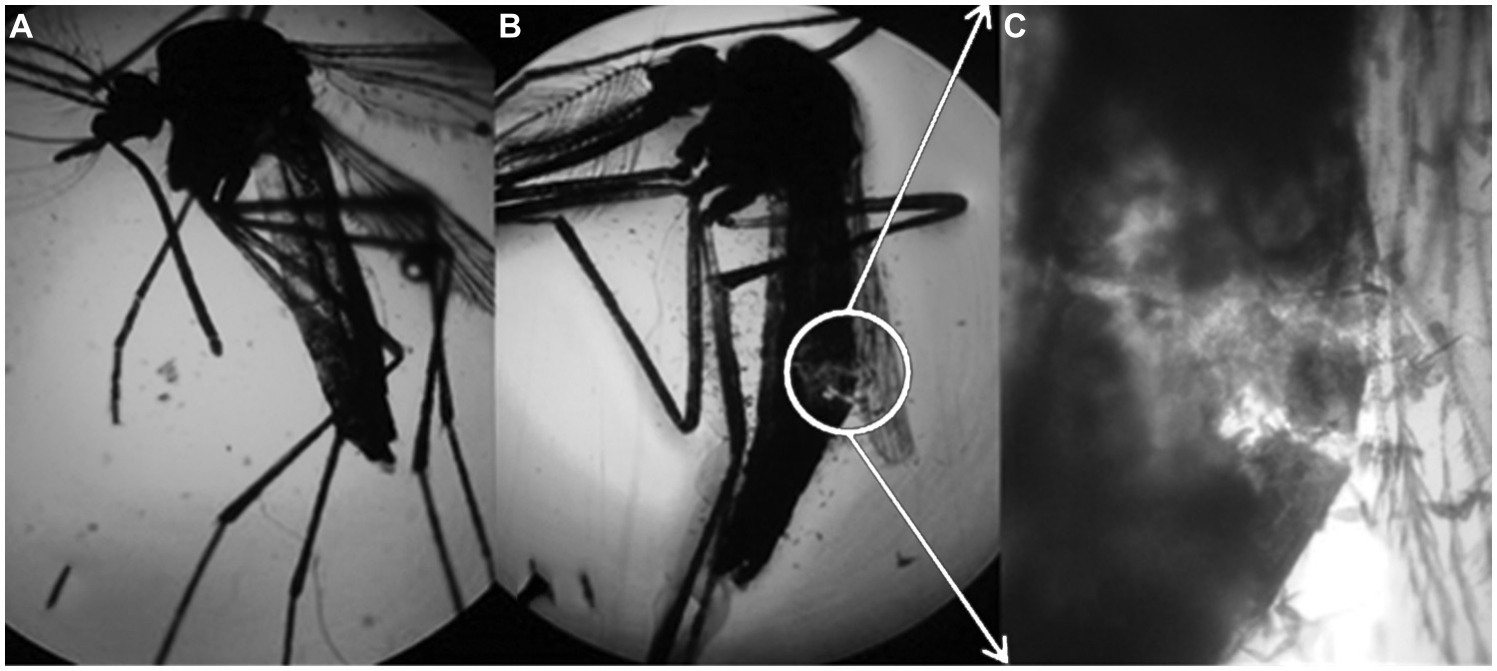
**Aedes aegypti subjected to the control treatment (enhanced 50x), (B) A. aegypti subjected to the solution containing 1000 mg/L of rhamnolipids (enhanced 50x) and (C) expansion of figure (B) enhanced 100x**.

Rhamnolipids are thought to disrupt the cuticle of the mosquito, as shown in **Figure 5**.

## DISCUSSION

The rhamnolipids solutions evaluated in this study have surface tension values ranging between 31.4 and 38.7 mNm^-1^. As a result, the larvae experience difficulties at the water/air interface, which reduces their respiratory efficiency and increases the number of attempts to stay at the surface. Lack of maintaining the correct siphon position during gas exchange was the cause of *A. aegypti* larval deaths. This is in agreement with that reported by Christophers (1960).

The air pockets in the trachea and tracheal trunks of *A. aegypti* larvae have a specific gravity close to that of water, which facilitates their ascent to the surface (Christophers, 1960).

The apex of the respiratory siphon is strongly hydrophobic, and secretions produced by the peri-spiracular glands are responsible for forming a non-wetting region when the structure is open and in contact with the surface (Christophers, 1960). Rhamnolipids likely interfere in this hydrophobic region, changing the wettability of the respiratory siphon and allowing water to flow in from the spiracular opening. Consequently, the hydrostatic balance is altered, which leads to difficulty returning to the surface. Furthermore, there is a greater energetic cost during transit due to the need for active swimming.

According to Davis et al. (2013), compounds produced by microorganisms are related to the feeding behavior of insects and may be powerful repellents. Verhulst et al. (2009) reported that odors produced by *P. aeruginosa* are not attractive to the mosquito species *Anopheles gambiae*.

The present study raises the hypothesis that the characteristic odor of rhamnolipids is recognized by *A. aegypti* adults as being unfavorable as a food source, thus leading to repellent behavior.

Rhamnolipids decreased the incidence of adult mosquitoes landing on mice and reduced the number of biting attempts.

Rhamnolipids led to breakup of the mosquito cuticle, leading to death. According to Kim et al. (2011), di-rhamnolipids act by thinning the cuticle in aphids. Dehydration of adjacent cell membranes also occurs, which separates cellular components and results in death.

Kamal et al. (2012) reported insecticidal activity of rhamnolipids against *Rhyzopertha dominica* and hypothesized that this biosurfactant acts on cuticular waxes and inter segmental membranes, which may also occur in *A. aegypti*.

Based on the results obtained in this study, rhamnolipids can replace currently used insecticides (e.g., temephos and pyrethroids). In addition to *A. aegypti* resistance against these insecticides, these chemicals are harmful to the environment and can lead to human health problems.

On the contrary rhamnolipids have been reported to be environmentally friendly and have low toxicity, which makes them suitable substitutes for chemical insecticides.

The rhamnolipids produced by *P. aeruginosa* LBI 2A1 have insecticidal activity against larvae and adults of *A. aegypti* and can be used as a repellent against mosquitoes. Therefore this microbial metabolite may be a new weapon to combat dengue and other arboviruses transmitted by *A. aegypti.*

## Conflict of Interest Statement

The authors declare that the research was conducted in the absence of any commercial or financial relationships that could be construed as a potential conflict of interest.
